# Fermentation and germination improve nutritional value of cereals and legumes through activation of endogenous enzymes

**DOI:** 10.1002/fsn3.846

**Published:** 2018-10-16

**Authors:** Smith G. Nkhata, Emmanuel Ayua, Elijah H. Kamau, Jean‐Bosco Shingiro

**Affiliations:** ^1^ Department of Agriculture Extension Services Lilongwe Malawi; ^2^ Department of Food Science and Nutrition University of Eldoret Eldoret Kenya; ^3^ Rwanda Agriculture and Animal Resources Board Kigali Rwanda

## Abstract

Cereals and legumes are outstanding sources of macronutrients, micronutrients, phytochemicals, as well as antinutritional factors. These components present a complex system enabling interactions with different components within food matrices. The interactions result in insoluble complexes with reduced bioaccessibility of nutrients through binding and entrapment thereby limiting their release from food matrices. The interactions of nutrients with antinutritional factors are the main factor hindering nutrients release. Trypsin inhibitors and phytates inherent in cereals and legumes reduce protein digestibility and mineral release, respectively. Interaction of phytates and phenolic compounds with minerals is significant in cereals and legumes. Fermentation and germination are commonly used to disrupt these interactions and make nutrients and phytochemicals free and accessible to digestive enzymes. This paper presents a review on traditional fermentation and germination processes as a means to address myriad interactions through activation of endogenous enzymes such as α‐amylase, pullulanase, phytase, and other glucosidases. These enzymes degrade antinutritional factors and break down complex macronutrients to their simple and more digestible forms.

## 1 INTRODUCTION

Processing of agricultural products remains the most important food and nutrition security aspect in the modern world. Due to urbanization, food is produced in remote areas and transported into towns or cities to feed the ever‐growing population. The seasonality of agricultural produce also necessitates processing of products so that they are available throughout the year. Processing of agricultural products is done to improve consumer acceptability while retaining its nutritional value. Different techniques are used for processing cereals and legumes that include fermentation and germination. Most processing techniques are localized to a certain region, while others are practiced across the world. For example, fermentation and malting are common practice in developing countries of Africa and South America, while nixtamalization is a common practice in Mexico. Fermented foods such as “ogi,” produced by acid fermentation of sorghum, millet, or maize, are widely consumed in West Africa (Omemu, 2011), while “chicha” and “masa” are common fermented foods made from fermented maize widely consumed in South American countries (Chaves‐Lopez et al., 2014). In central and southern Africa, “nshima” is made from fermented maize flour. While these techniques are important in improving the shelf life, palatability, and transportability, they can also have adverse effects on the nutrient profiles of these foods. Overall, it appears that some techniques such as fermentation and malting can simultaneously reduce antinutritional factors and enhance nutrient availability (Hotz & Gibson, 2007). Since fermentation and germination are widely used for processing cereals and legumes which constitute a large part of diets for households in developing countries, here we provide a review of how these techniques influence nutrient content and availability.

## 2 FERMENTATION

Fermentation is a desirable process of biochemical modification of primary food matrix brought about by microorganisms and their enzymes (Kahajdova & Karovicova, 2007). Fermentation is used to enhance the bioaccessibility and bioavailability of nutrients from different crops including maize (Hotz & Gibson, 2007) and improves organoleptic properties as well as extending the shelf life (Chaves‐Lopez et al., 2014; Li, Tayie, Young, Rocheford, & White, 2007; Steinkraus, 1994). It makes food safe by not only inhibiting growth of pathogenic bacteria due to antimicrobial activity of lactic acid (Li et al., 2007; Sahlin, 1999), but also detoxifies aflatoxin (Chaves‐Lopez et al., 2014).

With these desirable benefits, fermentation has been considered as an effective way to reduce the risk of mineral deficiency among populations, especially in developing countries where unrefined cereals and/or pulses are highly consumed (Kumar, Sinha, Makkar, & Becker, 2010). Unfortunately, it is also associated with proliferation of microorganisms such as yeast and molds that may cause food safety concerns (Omemu, 2011), reduction in provitamin A and antioxidant carotenoids (Ortiz, Nkhata, Buechler, Rocheford, & Ferruzzi, 2017), as well as loss of vitamins and minerals (Hotz & Gibson, 2007).

## 3 EFFECT OF FERMENTATION ON NUTRIENTS AND MINERALS

### 3.1 Carbohydrates

The major carbohydrate in cereals and legumes is starch which provides the most calories in developing countries (Chaves‐Lopez et al., 2014). Fermentation activates starch‐hydrolyzing enzymes such as α‐amylase and maltase which degrade starch into maltodextrins and simple sugars (Osman, 2011), respectively. Studies have shown increase in glucose during early stages of fermentation due to starch‐hydrolyzing effect of activated maltase and α‐amylase (El‐Hag, El‐Tinay, & Yousif, 2002; Osman, 2011). The glucose released during fermentation is a preferred substrate for microorganisms fermenting the food and could partly explain the decrease in total carbohydrate after 24 hr of fermentation (Osman, 2011). When both glucose and fructose were present during fermentation of pearl millet, microorganisms preferred glucose to fructose as a source of energy since the level of fructose remained constant. In addition, fermentation reduced starch content in millet varieties with subsequent increase in carbon dioxide and ethanol production throughout fermentation period. Moreover, pH was significantly reduced which activated phytase enzyme (El‐Hag et al., 2002).

### 3.2 Protein

The effect of fermentation on proteins has yielded inconsistent results likely due to different experimental designs, study durations, and variation in the initial protein or amino acid profile of foods. Several studies had reported increase (Chaven & Kadam, 1989; Doudu, Taylor, Belton, & Hamaker, 2003; El‐Hag et al., 2002; Pranoto, Anggrahini, & Efendi, 2013), while others observed decrease (Osman, 2011; Pranoto et al., 2013) in protein and/or some amino acids upon fermentation. It appears that most of these effects may not reflect actual changes but relative changes due to loss of dry matter as a result of microorganisms hydrolyzing and metabolizing carbohydrates and fats as source of energy. Fermentation of pearl millet for 24 hr increased protein content due to loss of carbohydrates (Osman, 2011). Lysine, glycine, and arginine were reduced (Osman, 2011), while methionine was increased (Chaven & Kadam, 1989) after fermentation. While increase in protein may partly be attributed to loss of dry matter during fermentation, bacterial fermentation is known to increase lysine content in fermented grains (Hamad & Fields, 1979). Bacterial fermentation produced lysine and increased its concentration by many folds and made cereal protein complete (Hamad & Fields, 1979). This increase may partly be due to degradation of complex protein by microorganism thereby releasing peptides and amino acids (Pranoto et al., 2013). However, it is reported that fermenting microorganisms also uses amino acid which could lower the protein content and quality of some fermented food (Osman, 2011; Pranoto et al., 2013).

Fermentation increases the digestibility of plant proteins (Ali, El‐Tinay, & Abdalla, 2003; Alka, Neelam, & Shruti, 2012; El‐Hag et al., 2002; Pranoto et al., 2013). Plant protein has poor digestibility relative to animal protein. Poor protein digestibility may cause gastrointestinal upset which may result in fecal excretion of protein. Hence, increased protein digestibility could reduce the levels of undigested proteins which can potentially cause food allergies due to poor absorption in the gut (Untersmayr & Jensen‐Jarolim, 2008). Combination of fermentation with other processing methods has more advantages. For example, fermentation followed by cooking was effective in increasing the digestibility of grain protein, bringing it nearly to the same level as meat(Khetarpaul & Chauhan, 1990; Osman, 2004; Yousif & El Tinayi, 2001, 2003) likely due to not only destruction of protease (trypsin) inhibitors (Khetarpaul & Chauhan, 1990; Osman, 2011) but also partial predigestion of grain proteins by bacteria during fermentation (Day & Morawicki, 2018). There is also reduction in tannins, oxalate, phytic acid, and carbohydrates which can complex with proteins and hence limiting accessibility by digestive enzymes (El‐Hag et al., 2002; Hassan, Yusuf, Adebolu, & Onifade, 2015; Osman, 2011; Sindhu & Khetarpaul, 2001).

More improvement in protein digestibility by fermentation is due to partial breakdown of complex storage protein into more soluble forms (Chavan, Chavan, & Kadam, 1988). Since the effectiveness of fermentation depends on activation of phytase, it is not surprising that fermenting roasted or cooked grains does not reduce phytic acid significantly as roasting or cooking destroy phytase (Egli, Davidsson, Juillerat, Barclay, & Hurrell, 2002). Furthermore, the degree of phytic acid degradation depends on the starting amount of phytase in the grain as grains with low phytase amounts such as corn, rice, oats, and millet require either a longer fermentation time or the addition of high‐phytase grains to significantly reduce phytates (Egli, Davidsson, Juillerat, Barclay, & Hurrell, 2003).

Fermentation can be done using starter culture or naturally. Due to lack of specificity, natural fermentation is less effective and nonpredictable but is most common form of fermentation in developing countries. Pranoto et al. (2013) compared the effect of *Lactobacillus plantarum* and natural fermentation for 36 hr on protein digestibility of sorghum flours using in vitro models. Protein digestibility was increased by 92% and 47% using *L*. *plantarum* and natural fermentation, respectively. This increase was attributed to increased proteolytic enzymes in *L*. *plantarum* that can not only degrade tannins which complex with proteins but also break down complex proteins thereby liberating more peptides and amino acids. In fact, Doudu et al. (2003) have previously reported that *L*. *plantarum* possess tannase that can cleave the protein–tannin complex thereby liberating proteins. Unfortunately, fermenting microflora can also utilize amino acids and protein during fermentation resulting in loss of amino acid and proteins (Pranoto et al., 2013). Therefore, it remains unclear on the optimum conditions for fermentation that could lead to maximum protein digestibility with minimal loss of protein (Table 1).

### 3.3 Minerals

**Figure 1:**
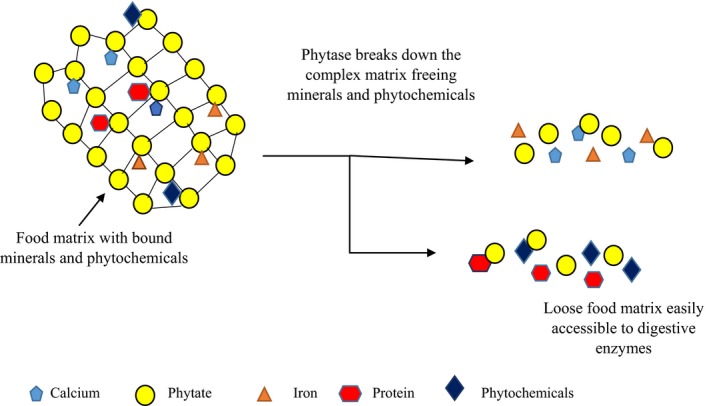
Plausible mechanism by which fermentation leads to increased minerals, phytochemicals, and proteins bioavailability

Cereals and legumes are the major sources of minerals in developing countries where they are widely consumed. Minerals from plant sources have very low bioavailability because they are found complexed with nondigestible material such as cell wall polysaccharides (Torre, Rodriquez, & Saura‐Calixto, 1991) as well as phytate. Notably, potassium is integral part of phytate molecules where it is covalently bonded rendering it inaccessible by digestive enzymes. The complex matrices in which these minerals are entrapped and bonded (Figure 1) are largely responsible for their low bioavailability. Fermentation is one of the processing methods that are applied to free these complexed minerals and make them readily bioavailable (Lopez, Gordon, & Fields, 1983; Pranoto et al., 2013).

Fermentation increased magnesium, iron, calcium, and zinc content in some fermented foods that are commonly consumed in India and associated with the decrease in the amount of phytates (Pranoto et al., 2013). However, the increase in mineral content might be due to loss of dry matter during fermentation as microbes degrade carbohydrates and protein (Day & Morawicki, 2018). Fermentation also increases bioavailability of calcium, phosphorous, and iron likely due to degradation of oxalates and phytates that complex with minerals thereby reducing their bioavailability (Sripriya, Antony, & Chandra, 1997).

There are different mechanisms by which fermentation increases the mineral bioavailability. Firstly, fermentation reduces phytic acid that binds minerals making them free and more available (Lopez et al., 1983). However, this effect is counteracted by release of tannins during fermentation especially in high‐tannin cereals such as sorghum (Osman, 2011; Sripriya et al., 1997). The increase in tannin during fermentation was attributed to hydrolysis of condensed tannins such as proanthocyanidin to phenols (Emambux & Taylor, 2003; Sripriya et al., 1997). Tannins bind minerals and reduce their bioavailability (Emambux & Taylor, 2003) depending on the duration of fermentation. Prolonged fermentation decreased the tannin due to microbial phenyl oxidase action (Emambux & Taylor, 2003). However, transformation of tannins to phenols occurring during fermentation increases phenol content that interacts with minerals leading to an inhibition of mineral bioavailability (Sripriya et al., 1997), and this is a potential reason why degradation of phytates in sorghum with high‐tannin content does not increase in vitro bioaccessibility of iron (Mohite, Chaudhari, Ingale, & Mahajan, 2013). Secondly, fermentation loosens the complex matrix that embeds minerals. Both phytase and α‐amylase make the matrix loose by degrading phytate and starch, respectively. Moreover, some fermenting microorganisms have the ability to degrade fiber which loosen the food matrix further (Liang, Han, Nout, & Hamer, 2008). Therefore, the effect of fermentation depends on food composition and that other food components such as dietary fibers may slow down the accessibility of some mineral. To offset these challenges, germination or incubation of foods with polyphenol oxidase (PPO) or phytase during fermentation may help reduce the tannins or phytates, respectively, and thus make minerals bioaccessible (Towo, Matuschek, & Svanberg, 2006). Nonetheless, such processes alone may not be sufficient to reduce the antinutritional factors significantly but may be prerequisite steps to fermentation. In fact, one study demonstrated that genetic modification of sorghum high in tannin is not sufficient to increase iron bioaccessibility but rather a combination of genetic modification and fermentation processes (Kruger, Taylor, John, & André, 2012). Thirdly, low pH obtained during fermentation increases iron absorption due to conversion from ferrous iron, which is less absorbable, to ferric iron, which is readily absorbed. Moreover, fermentation provides optimum pH for enzymatic degradation of phytate. When fermentation is preceded by grinding, mineral bioavailability is further improved. This is because grinding increases grain surface area and breaks up cellular structure, thereby releasing phytase that degrades phytate (Egli et al., 2003; Hemalatha, Platel, & Srinivasan, 2007; Leenhardt, Levrat‐Verny, Chanlia, & Eameasy, 2005; Reale, Konietzny, Coppola, Sorrentino, & Greiner, 2007).

### 3.4 Phytochemicals

For a long period, the importance of phytochemicals (phytonutrients) to human nutrition and health was not well known. Phytochemicals are important plant secondary metabolic products produced in phenylpropanoid biosynthesis and shikimate pathways during the growth of plants (Zhang, Xu, Gao, Huang, & Yang, 2015). During growth, L‐phenylalanine, under the effect of phenylalanine ammonia lyase (PAL) catalyzation, changes into cinnamic acid. From then on, many phenolic components such as caffeic acid, ferulic among others are synthesized. These can later be converted into tannins, flavonoids, lignins, and other compounds. Advances in research have revealed importance of these phytonutrients to human health by virtue of their antioxidant properties (Zhang et al., 2015), cholesterol‐lowering effect (Golzarand, Mirmiran, Bahadoran, Alamdari, & Azizi, 2014; Gunness & Gidley, 2010), and reduction in the production of pro‐inflammatory cytokines and immunosuppressive cells (Lesinski et al., 2015). Due to the complexity of studying phytochemicals, few studies have focused on studying the effects of traditional processing techniques due to limited capacity in most laboratory studying these traditional methods. Nevertheless, fermentation has significant effect on phytochemicals that are both beneficial and adverse. Fermentation of high‐carotenoid biofortified maize resulted in significant loss of carotenoids (Li et al., 2007; Ortiz et al., 2017) depending on the duration of fermentation process (Ortiz et al., 2017). Fermentation of biofortified maize for 24 and 72 hr retained 60%–100% of provitamin A carotenoids. However, after 120 hr of fermentation, retention significantly decreased to between 27% and 48% depending on genotypes (Ortiz et al., 2017).

Fermentation for 120 hr significantly reduced in vitro bioavailability of carotenoids in six high‐carotenoid biofortified maize genotypes (Ortiz et al., 2017). A number of mechanisms have been proposed explaining observed low bioavailability of carotenoids from fermented maize. Due to disruption of matrix possibly by activated endogenous enzymes and microorganism, there is an increase in concentration of calcium which might enhance saponification of free fatty acids leading to reduced fat absorption and increase in fatty acid excretion in feces (Lorenzen et al., 2007). Fatty acids are very critical during absorption of lipophilic carotenoids. However, some reports indicate that fermentation improves β‐carotene absorption in rats due to disruption of food matrix (Phorbee, Olayiwola, & Sanni, 2013). It is therefore difficult to make conclusion based on these findings because they used different models and sources of β‐carotene. β‐Carotene bioavailability is dependent on genotype and processing method used to develop test food (Ortiz et al., 2017; Phorbee et al., 2013).

The effects of fermentation on phytonutrients have been examined in soybeans (Hubert, Berger, Nepveu, Paul, & Dayde, 2008), and other cereals or pseudocereals (Dordevic, Marinkovic, & Dimitrijevic‐Brankovic, 2010; Oghbaei & Prakash, 2016; Wang, Wu, & Shyu, 2014). The focus has been freeing phytochemicals by fermentation (Hubert et al., 2008) as some of them can interact with proteins, carbohydrates, or minerals making them unavailable (Doudu et al., 2003; El‐Hag et al., 2002). Moreover, the microorganisms fermenting the foods can utilize these phytochemicals thus leading to their reduction (El‐Hag et al., 2002; Hubert et al., 2008). For example, Hubert et al. (2008) witnessed a decrease in phytosterols, glycosylated soyasaponins, and tocopherols when soybean germs were fermented for 48 hr using strains of lactic acid bacteria. In this study, amounts of phytosterol were reduced from 4.2 mg/g at the beginning of the study to 1.1 mg/g at the end of the study. These authors suggested that the decrease in glycosylated soyasaponins could be due to their conversion from their conjugation, 2,3‐dihydroxy‐2,5‐dihydroxy‐6‐methyl‐4H‐pyran‐4‐one (DDMP) to non‐DDMP forms. Soyabeans are rich sources of isoflavones such as genistein, daidzein, and glycitein which are potent antioxidants. Fermentation was reported to reduce isoflavones significantly due to hydrolysis of glucosides into aglycone (Manach, Scalbert, Morand, Remesy, & Jimenez, 2004).

The effect of fermentation on phytonutrients is not specific. Wang et al. (2014) investigated the effect of fermentation on antioxidant profiles of four cereals using *Bacillus subtilis* and *L. plantarum*. There was a significant increase in the total phenolic acid and total flavonoid contents with greatest increase in samples with starter culture. Dordevic et al. (2010) demonstrated that *Lactobacillus rhamnosus* was more effective than *Saccharomyces cerevisiae* in releasing total phenolics during fermentation of cereals. During fermentation, microorganisms break down cereal grain matrices leading to release of bound phytochemicals (Dordevic et al., 2010). *L. plantarum* and *B. subtilis* have been previously reported to possess β‐glucosidase that can cleave glucoside bonds between phytochemicals and sugars thereby releasing phytochemicals (Duenas, Fernandaz, Hernandez, Estrella, & Munoz, 2005; Kuo, Cheng, Wu, Huang, & Lee, 2006). Thus, the ability of fermentation to increase antioxidant properties of foods can be explored as a cost‐effective way to reduce oxidative stress within the body after consuming such foods.

### 3.5 Fermentation and glycemic index

Fermentation has dual effect on glycemic index (GI). Some studies have reported increased (Ihediohanma, 2011; Ihekoronye & Ngoody, 1985) while others have reported decreased (Mlotha, Mwangwela, Kasapila, Siyame, & Masamba, 2016; Scazzina, Del Rio, Pellegrini, & Brighenti, 2008) GI after consumption of fermented foods. The low GI of fermented food has been attributed to the short‐chain organic acids produced during fermentation such as lactic acid, acetic acid, and propionic acid (Ostman, Granfeldt, Persson, & Bjorck, 2005). Eating lactic acid fermented foods reduced postprandial blood glucose spike (Ostman, Nilsson, Liljeberg Elmstah, Molin, & Bjorck, 2002; Scazzina et al., 2008). The mechanism for the glucose‐lowering action of lactic acid has been suggested to be due to a lowered rate of starch hydrolysis in the upper small intestine (Ostman et al., 2002) suggesting that lactic acid may reduce activity of starch‐hydrolyzing enzymes. Unlike lactic acid, the mechanism of action for propionic and acetic acids appears to be a lowered rate of gastric emptying and suppressing enzymatic activity (Darwiche, Almer, Bjorgell, Cederholm, & Nilsson, 2001; Liljeberg & Bjorck, 1998). This perhaps could be the reason why sourdough bread has been linked to lower postprandial glucose level and improve glucose response in healthy subjects because organic acid produced by sourdough microflora delay gastric emptying without influencing starch accessibility or general bioavailability (Scazzina et al., 2008). Ostman et al. (2005) also observed a significant decrease in GI when vinegar was supplemented in bread meal in a dose‐dependent manner. Compared with the reference meal, the highest level of vinegar significantly lowered the blood glucose response at 30 and 45 min, the insulin response at 15 and 30 min postprandially (Ostman et al., 2005). The low and intermediate levels of vinegar also lowered the 30 min glucose and the 15 min insulin responses significantly compared with the reference meal. In contrast, lactic acid in fermented milk did not lower GI in healthy volunteers (Ostman, Liljeberg‐Elmstah, & Bjorck, 2001). Based on these observations, it is logical to think that natural fermentation of starch and sugars by yeast starter culture produces lactic and propionic acids that reduce amount of glucose that could be released from food thereby lowering the GI of the food.

**Table 1:** Summary of the effect of fermentation and germination on nutritional value of different cereals and legumes

Processing technique	Cereal/Legume	Outcomes	References
Germination (malting/sprouting)	Buckwheat	Increased phenolic compounds, reducing sugars, flavonoids, crude protein, and antioxidant activities Decreased phytic acid, trypsin inhibitor activity (TIA), and crude fat	Zhang et al. (2015), Zheng et al. (2006)
Maize	Increased total soluble, free and conjugated phenolics, protein, and niacinDecreased fat Increased crude fiber and total protein content	Zilic et al. (2015), Ongol, Nyozima, Gisanura, and Vasanthakaalam (2013)
Australian sweet Lupin Brown rice Peas	Increased fat Decreased Phytic acid Decreased dietary fiber	Rumiyati et al. (2012), Liang et al. (2008), Martin‐Cabrejas et al. (2003), Chinma, Anuonye, Simon, Ohiare, and Danbaba (2015)
Finger millet	Increased sugars and protein digestibility Increased TIA, tannins, phytates, and starch	Mbithi‐Mwikya et al. (2000)
Sorghum and millet	Increased crude fiber, minerals, protein digestibility, sucrose, glucose, fructose, and α‐amylase activity Decreased sucrose, TIA, oxalates, tannins, and phytates Increased minerals, vitamin content, and fiber	Ogbonna et al. (2012), Ojha et al. (2018), Onyango et al. (2013), Traore et al. (2004)
Ragi Foxtail millet, wheat, andchickpea	Increased protein, carbohydrate, crude fiber, vitamin C, and iron Decreased fat	Desai et al. (2010), Laxmi et al. (2015)
Lentils and faba beans (6 days, 20 Oc)	Reduced thiamine amounts Increased riboflavin and niacin	Wang et al. (2014), Prodanov et al. (1997)
Rice (24 hr, 28–30°C)	Germination increased crude proteins, niacin, free amino acids, and α‐tocopherol Ash, crude fat, and carbohydrate were unchanged	Moongngarm & Saetung (2010)
Sorghum (7 days)	Increased ash and protein but with reduction in moisture Increased amylopectin, water absorption index, but reduced amylose	Otutu et al. (2014)
Foxtail millet, wheat, and chickpea (steeping in lime solution (0.05%) for 12–48 hr at 20°C, germination for 3–5 days)	Increase in protein content	Laxmi et al. (2015)
White sorghum, red sorghum, pearl millet (steeping for 24 hr at 25°C for 24 hr and germination for 72 hr at 25°C)	Decrease in polyphenols, tannin, and phytates Increase in protein digestibility	Onyango et al. (2013)
White sorghum (steeping for 8–48 hr, germination for 24–96 hr)	Decrease in protein content Decrease in carbohydrates Increase in sodium, potassium, phosphorous, calcium, magnesium	Ogbonna et al. (2012)
Green gram, cowpea, lentil, chickpea (Soaking in water for 12 hr at 22–25°C, and germination for 24 hr)	Increase in protein Decrease in antinutrients: oxalate, tannin, trypsin inhibitor, and phytates Decrease in iron, calcium, and phosphorous Improved starch and protein digestibility	Ghavidel and Prakash (2007)
Red kidney beans (Soaking in water for 6 hr at room temperature, germination for 4 days at 22°C)	Decrease in cyanide, tannins, polyphenols, and phytic acid	Yasmin et al. (2008)
Kidney, mung beans, soybean, and peanuts (soaking in water for 6 hr and germination until emergence of radical at maximum 5 mm)	Increase in total sugars Increase in total dietary fibers	Megat et al. (2016)
Finger millet (96 hr at 25°C) Oat seeds (24 to 144)	Decrease in total carbohydrates Increase in protein Decrease in starch Increase in free sugars	Nirmala et al. (2000), Tian et al. (2010)
Flax seeds (8 days)	Oleic, linoleic, and linolenic were unchangedIncreased phosphatic acids, FFA, and lysophosphatidylcholine Increased lipase activity but reduced total lipids	Wanasundara et al. (1999)
Barley	Reduced β‐glucans while β‐glucanase increased	Wang et al. (2014)
Fermentation	Adlay, walnut, chestnut, and lotus seed	Increased flavonoid and phenolic extract increased scavenging of DPPH radicals Enhanced inhibition of LPS production mediated by reactive oxygen species (ROS) in cells	Wang et al. (2014)
Sorghum (24 hr)	Reduced phytic acid, trypsin inhibitors, and tannins Increased in vitro protein digestibility	Osman (2004)
Sorghum (36 hr)	Increased titratable acidity, crude protein, protein digestibility, and total solids	Yousif and El Tinayi (2001)
Cocoa (6 days)	Reduced antioxidant capacity and polyphenols contents	Albertini et al. (2015)
Pearl millet (4 hr)	Increase in glucose Decrease in total carbohydrates Decrease in AIA No change in fructose	Osman (2011)
Pearl millet (24 hr)	Increase in total protein Decrease in specific amino acids such as lysine, glycine, and arginine	Osman (2011)
Wheat, barley, rice, and maize	Increase in lysine content	Hamad and Fields (1979)
Wheat, barley, rice, millet, and maize (22–25°C) and 37°C	Increase in available lysine	Chaven and Kadam (1989)
Pearl Millet	Reduction in trypsin inhibitors Increased protein digestibility	El‐Hag et al. (2002)
Finger millet	Increased bioavailability of calcium, phosphorus, and iron	Sripriya et al. (1997)
High‐carotenoid biofortified maize	Loss of carotenoids with modest losses after 24 and 72 hr but bigger losses after 120 hr. Reduced bioavailability	Ortiz et al. (2017)
Soybeans (48 hr)	Decrease in phytosterols, glycosylated saponins, and tocopherols	Hubert et al. (2008)
Wheat flour made into sourdough bread	Decreased glycemic index	Scazzina et al. (2008)
Vinegar‐supplemented bread	Decreased glycemic index	Ostman et al. (2005)

*Note*

FFA: free fatty acids; DPPH: 2,2‐diphenyl‐1‐picrylhydrazyl; LPS: lipopolysaccharide; AIA: amylose inhibitor activities; TIA: trypsin inhibitor activity.

**Figure 2:**
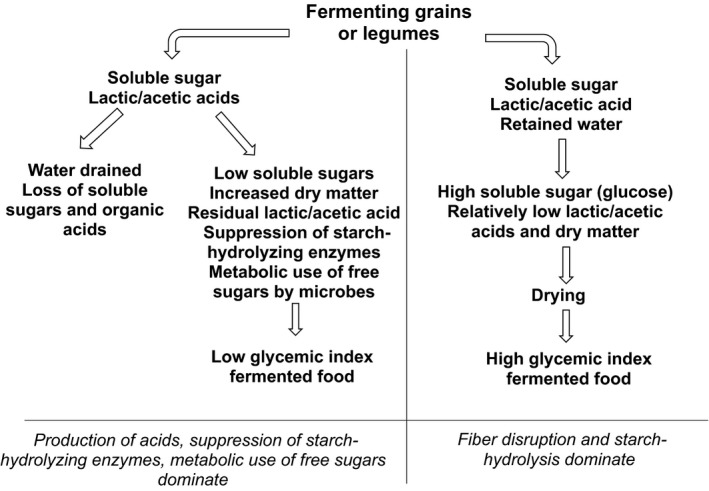
Schematic diagram illustrating how fermentation results into different GI of fermented food. GI: glycemic index

There is still gap on scientific knowledge on the increase in GI due to fermented cereal and legumes products. However, GI increased when subjects consumed 50 g of fermented garri made from cassava (Ihediohanma, 2011) with the variation dependent on fermentation time. The increase was likely due to ease of digestion and absorption of glucose as a result of degradation of fiber by microorganisms during fermentation. Ihekoronye and Ngoody (1985) stated that maltose is formed during fermentation of starch which is further converted to D‐glucose when hydrolyzed in aqueous solution in the order starch–dextrin–maltose–glucose. It is, therefore, hypothesized that by the same mechanism, fermentation of cereals and legumes might have similar effect; hence, more studies are needed. Increase in fermentation period may bring about release of more glucose and subsequently increase postprandial glycemic response. Nevertheless, the effect of fermentation largely depends on acids produced, starch hydrolysis, and disruption of fiber. Whichever factor dominates determines the effect of fermentation on GI of food. When more acids are produced, fermented food has lower GI due to lowered rate of gastric emptying and suppression of starch‐digesting enzymes (Darwiche et al., 2001; Liljeberg & Bjorck, 1998). When disruption of fiber and hydrolysis of starch dominates, GI increases due to ease of release of glucose (Ihediohanma, 2011; Ihekoronye & Ngoody, 1985) (Figure 2).

## 4 GERMINATION OR MALTING

Malting is a process where cereals are steeped and then germinated. After germination, the seeds are then matured (fermented) by storing away from the sun (Hotz & Gibson, 2007). Germination is the process occurring at the beginning of the development of seeds into plants, during which they sprout (Rumiyati, James, & Jayasena, 2012). It involves changes in the nutritional, biochemical, and sensory characteristics of the food. It is used in processing of cereals to improve nutritional quality as it results in reduction of antinutritional factors (Laxmi, Chaturvedi, & Richa, 2015; Oghbaei & Prakash, 2016; Onyango et al., 2013). These changes are greatly associated with the activation of some endogenous enzymes making germinated foods higher in nutritional quality compared to nongerminated seeds (Zhang et al., 2015). Additionally, many African societies traditionally used malting as a processing method in the manufacture of alcoholic drinks (Taylor & Dewang, 2001).

## 5 EFFECT OF GERMINATION OR MALTING ON NUTRIENTS AND MINERALS

### 5.1 Carbohydrates

The effect of malting and germination on carbohydrates is largely dependent on activation of hydrolytic and amylolytic enzymes which results into decrease in starch and increase in simple sugars in a time‐dependent manner. Germination and malting facilitate the enzymatic breakdown of carbohydrates into simple sugars through activation of endogenous enzymes such as α‐amylase thereby improving digestibility (Oghbaei & Prakash, 2016) as a result of degradation of starch to provide energy for the seed development (Zhang et al., 2015). Germinating white sorghum for 24 and 36 hr resulted into a decrease in carbohydrates and increase in simple sugars (Obizoba & Atii, 1991). Both germination and malting increased activity of α‐amylase (Traore, Mouquet, Icard‐Verniere, Traore, & Treche, 2004) and consequently increased the digestibility of starch, making it a good method in the preparation of complementary and infant foods (Desai, Kulkarni, Sahoo, Ranveer, & Dandge, 2010; Svanberg & Lorri, 1997). Another study on in vitro digestibility of green gram, cowpea, lentil, and chickpea reported increased digestibility of starch by 53%–82% after germination for 24 hr (Ghavidel & Prakash, 2007).

A decline of starch content is initiated by malting conditions allowing enzymatic activity of amylase and pullulanase to hydrolyze starch into smaller sugar molecules such as maltotriose and maltose. Following malting of finger millet for 96 hr, the total carbohydrates and starch content dropped from 81% to 58% and 65% to 43%, respectively (Nirmala, Subba Rao, & Muralikrishna, 2000). In oat seeds, germination for 24–144 hr reduced starch content from 60% to 21%, while free sugars increased from 5% to 28% (Tian et al., 2010). Germination of kidney, mung beans, soybean, and peanuts showed an increase in total sugars by 14%, 22%, 19%, and 26%, respectively (Megat, Azrina, & Norhaizan, 2016). The same study also revealed an increase in total dietary fiber from 37% to 60% in kidney, 29% to 32% in mung beans, 32% to 73% in soybean, and 27% to 40% in peanuts (Megat et al., 2016).

The duration of the process is a significant factor in malting. The maximum hydrolysis of starch is between 48 and 72 hr when amylase activity is at maximum (Nirmala et al., 2000; Tian et al., 2010). At extended malting or germination, the enzymatic activity is slowed down. In sorghum, the enzymatic activity of glucosidases and pullulanases was low when malting went beyond 96 hr (Nirmala et al., 2000). The content of the reducing sugars in cereals and legumes was not significantly affected during the first 12 hr of germination. However, after 12 hr the content of reducing sugars increased 20 times suggesting increased enzymatic hydrolysis of starch (Zhang et al., 2015). This happens due to the action of α‐amylase which is activated during germination leading to hydrolysis of carbohydrates, change of taste, and digestibility of the carbohydrates (Zhang et al., 2015). Mbithi‐Mwikya, Camp, Yiru, and Huyghebaert (2000) noted a similar trend in germinated seeds. In earlier stage of germination, large portions of soluble sugars are expected to be used up during respiration and not enough α‐amylase has been synthesized or activated to hydrolyze starch, leading to less increase in sugars (Mbithi‐Mwikya et al., 2000). However, after 36–48 hr of germination, the dormancy is lost as the amylolytic enzymes synthesized in the aleurone layer migrate into the endosperm and initiate the hydrolysis of starch granules (Mbithi‐Mwikya et al., 2000). Glucose and fructose levels are generally low in the raw cereals. However, on germination, the two soluble sugars increase significantly such that their levels supersede that of sucrose activation of invertase which hydrolyzes sucrose into glucose and fructose during germination (Traore et al., 2004).

Germination could be an effective way of improving the fiber content in foods (Chinma, Adewuyi, & Abu, 2009; Jan, Saxena, & Singh, 2017; Rumiyati et al., 2012). Germination of lupin and peas increased fiber content by 456% and 100% on dry basis, respectively (Martin‐Cabrejas et al., 2003; Rumiyati et al., 2012). Loss of dry matter resulting from enzyme hydrolysis of starch and microbial breakdown of cellular materials such as proteins, fats, and carbohydrates could explain the increase in fiber observed in these studies. It may also result from the increase in the cellular structure of the plants as they germinated. The crude fiber consisting of cellulose, lignin, and hemicelluloses increase significantly during germination process (Laxmi et al., 2015; Zheng et al., 2006) as the plant cells synthesize different cellular components. The increase in fiber is desirable because dietary fiber slows down glucose release from food (Riccardi & Rivellese, 1991) which could be beneficial for people with diabetes. Moreover, fiber forms gels in the stomach that slows down starch digestion and gastric emptying which subsequently increase satiety (Yu, Ke, Li, Zhang, & Fang, 2014). Due to inability of both salivary and pancreatic α‐amylase to break down fiber, fiber reaches the colon where they are fermented by colonic bacteria to produce short‐chain fatty acids such as butyrate, acetate, and propionate which have other physiological function such as regulating satiety (Byrne, Chambers, Morrison, & Frost, 2015; McNabney & Henagan, 2017). Butyrate and acetate have been shown to upregulate genes involved in fatty acid oxidation (Canfora, Jocken, & Blaak, 2015), inducing lipolysis in adipocytes (Rumberger, Arch, & Green, 2014), which is important in initiating weight loss in overweight persons.

### 5.2 Proteins

Effect of germination and malting on protein seems to be conflicting. Protein has been reported to increase upon germination depending on type of grains/seed (Laxmi et al., 2015; Otutu, Ikuomola, & Oloruntoba, 2014). However, other scholars have reported a reduction in total proteins albeit with increase in specific amino acids such as lysine, tryptophan, and methionine in after germination of quinoa (Bhathal & Kaur, 2015). The increase in proteins may be due to loss of dry weight as some carbohydrates and fats are utilized during respiration but also some amino acids are synthesized during germination (Jan et al., 2017; Ongol et al., 2013). Moreover, protein losses during germination have been attributed to their degradation by proteases. Therefore, the actual protein content will be determined by net effect of synthesis and breakdown. Overall, it seems the net protein synthesis outweighs breakdown due to critical need for synthesis of nucleic acids required for growth, which can influence a net increase in proteins (Moongngarm & Saetung, 2010). After germination of buckwheat for 72 hr, protein content increased significantly (Zhang et al., 2015) probably due to higher rate of protein synthesis compared to proteolysis.

Germination also improves the biological value of proteins. Ghavidel and Prakash (2007) have showed that the in vitro digestibility of protein, crucial in determining the protein quality of food, was increased by a range of 14%–18% after germination of green gram, cowpea, lentil, and chickpea. The protein digestibility increased more when malting was combined with fermentation (Onyango et al., 2013). In vitro protein digestibility also increased after germination of maize and finger millet (Mbithi‐Mwikya et al., 2000; Ongol et al., 2013). Protein digestibility increased by 64% after germination of finger millet (Mbithi‐Mwikya et al., 2000) due to proteolysis and partial solubilization that comes with sprouting the seeds, as evidenced by increased water‐soluble proteins and free amino acids in the sprouted seeds (Mbithi‐Mwikya et al., 2000). Conflicting results exist indicating a decrease in protein content in white sorghum (Ogbonna, Abuajah, Ide, & Udofia, 2012) likely due to differences in germination conditions such as steeping, cultures used, and germination time.

Various cereals such as soybeans contain trypsin inhibitor. By inhibiting the activity of trypsin, it greatly compromises the body's ability to digest the ingested proteins. Trypsin inhibitor is quite a nutritional challenge because it is resistant to heat and remains stable at high temperatures. As such, many cooking methods can do little or nothing in reducing its activity. However, upon germination, activity of trypsin inhibitor is gradually reduced with a consequent rise in trypsin activity (Mbithi‐Mwikya et al., 2000; Zhang et al., 2015). Ikeda, Arioka, Fujii, Kusano, and Oku (1984) revealed that upon germination, the trypsin inhibitor rapidly decreases and, by the 4th day of germination, some seedlings have little or no quantities of trypsin inhibitor.

### 5.3 Minerals

One of the main reasons for processing foods is to make sure that their nutritional value is maintained over long periods and, where possible, improved. Phytic acid is one of the antinutritional factors common in cereals, which is responsible for binding minerals thus making them not readily bioavailable (Liang et al., 2008). A study carried out on buckwheat (Zhang et al., 2015) showed that phytic acid in buckwheat decreased with increase in the germination time due to activation of phytase which hydrolyzes phytic acid into phosphoric acid and myoinositol thereby making minerals more bioavailable (Liang et al., 2008; Mbithi‐Mwikya et al., 2000).

Mineral availability was grain specific with highest availability for iron in wheat, zinc in rice and wheat, manganese in rice and soybean, and calcium in soybean, rice, and faba beans (Luo, Xie, Jin, Wang, & He, 2014). The difference in mineral availability from different cereals and legumes after germination for similar period may be related to differences in phytate content, phytase activation, extent of binding of minerals within the matrix, or interaction of these factors. Malting of sorghum, foxtail, and chickpea significantly increased the content of sodium, potassium, phosphorus, calcium, and magnesium(Desai et al., 2010; Idris, AbdelRahaman, Elmaki, Babikar, & Eltinay, 2007; Laxmi et al., 2015; Ogbonna et al., 2012) but decreased calcium and iron (Desai et al., 2010; Laxmi et al., 2015; Ogbonna et al., 2012). This difference could be accounted for by different processing methods such as differing steeping times and freeing of bound minerals during malting (Onyango et al., 2013). Some cereals which contain high antinutritional factors such as tannins and phytates tend to have most of the trace elements bound. Foxtail millet and chickpea have higher phytate than wheat. On malting, more of the bound iron was released in foxtail millet and chickpea compared to wheat (Ogbonna et al., 2012). The increase could be due to leaching of the antinutritional factors that bind the minerals (Idris et al., 2007). It has been hypothesized that the remarkable increase in phytase activity during germination helps reduce phytic acids, which bind minerals subsequently leading to increased mineral availability (Luo et al., 2014). Legumes are rich in protease inhibitors, α‐amylase inhibitors, lectins, polyphenolic compounds, tannins, and phytic acid that cause poor absorption and digestibility of minerals and nutrients (Yasmin, Zeb, Khalil, Paracha, & Khattak, 2008). Importantly, legumes contain endogenous phytase enzyme that is activated by malting to destroy phytate (Luo et al., 2014; Ogbonna et al., 2012).

### 5.4 Vitamins

Germination increases various vitamins present in cereals and legumes such as tocopherols (α‐, β‐, and γ‐tocopherols), riboflavin (Vitamin B_2)_, and total niacin (Vitamin B_3_) (Kim et al., 2012) due to synthesis of these vitamins by the new sprouts (Zilic et al., 2015). However, losses of water‐soluble vitamins are common during germination. Losses of thiamine (Vitamin B_1_) were due to leaching in germinated brown rice (Moongngarm & Saetung, 2010). Nonsignificant increase in pyridoxine (Vitamin B_6_) and niacin in germinated brown rice was also observed. In another study where lentils and faba beans were germinated at 20°C for 6 days, there was a reduction in thiamine (Prodanov, Sierra, & Vidal‐Valverde, 1997) and increase in riboflavin and total niacin. Nevertheless, with prolonged germination, there was a trend of increase in thiamine content.

Vitamin C (ascorbic acid) content significantly increased in malted ragi, wheat, mung beans, chickpea, and wheat (Desai et al., 2010; Guo, Li, Tang, & Liu, 2012; Laxmi et al., 2015). Vitamin C can be synthesized by plants and animals from glucose, mannose, and galactose (Banhegyi & Mandl, 2001; Wheeler, Jones, & Smirnoff, 1998). Therefore, the increase in vitamin C during malting/germination is driven by enzymatic hydrolysis of starch by amylases and diastases that increase availability of glucose for the biosynthesis of vitamin C. It is this enhanced content of glucose that acts as a precursor to the formation of vitamin C (Desai et al., 2010). This postulation is supported by work done by Loewus, Kelly, and Hiatt (1960) who carried out a study on rats. They reported that D‐glucose is converted into D‐glucuronolactone which then changes to L‐gluconolactone and finally into L‐ascorbic acid. This study confirmed that C‐6 of glucose could be oxidized to form the carboxyl carbon of the ascorbic acid (Loewus et al., 1960). Taur, Pawar, and Ingle (1984) concluded that the same could happen in plants during fermentation or malting. This evidence, therefore, supports the notion that malting increases the ascorbic acid in cereals, improving their nutritional and antioxidant properties.

### 5.5 Fat

Lipid content of cereals slightly increases during the steeping stage of malting but later declines during the germination phase (Traore et al., 2004) as lipids are used for respiration process. Kim et al. (2012) observed an increase in crude lipids, linoleic acid, and oleic acid in germinated rice, while Moongngarm and Saetung (2010) did not find changes in fat content when rice grains were germinated. Other studies have reported that germination reduces fat content (Jan et al., 2017; Wanasundara, Wanasundara, & Shahidi, 1999) due to hydrolysis and utilization of fats as an energy source for biochemical reactions during germination (Chinma et al., 2009; Jan et al., 2017; Moongngarm & Saetung, 2010). The discrepancies between these studies could be related to differences in germination time and different sources of rice used in the study.

### 5.6 Phytochemicals

Germination of buckwheat increases total phenolics, flavonoid, and condensed tannin contents (Zhang et al., 2015). Zilic et al. (2015) also found that sprouting white, sweet, and yellow maize kernels for 5 days yielded an increase of 92%, 46%, and 50%, respectively, of bioavailable phenolic compounds. PAL, the enzyme that catalyzes the pathways responsible for biosynthesis of the different phytochemicals, is responsible for limiting the rate of biosynthesis of phenolic acids and flavonoids. Tang and Zhao (1998) concluded that the PAL activity is enhanced during the process of germination. Another possible explanation is that there could be hydrolysis of bound phenolic compounds as well as de novo biosynthesis of phenols in the embryonic axis of the sprouts (Hotz & Gibson, 2007; Onyango et al., 2013; Zilic et al., 2015). The increased content of phytochemicals results into increased antioxidant activity of germinated cereals and legumes.

Phytochemicals can negatively affect bioavailability of nutrients as already discussed (Liang et al., 2008; Mbithi‐Mwikya et al., 2000). On the other hand, prolonged soaking and fermentation can help reduce the content of these phytochemicals through leaching (Ogbonna et al., 2012). Microbial activity during fermentation process reduces tannin content as does the activity of the enzyme tannin acyl hydrolase (Ojha et al., 2018). Owing to the reduction in the tannin and phytic acid content in malted cereals, minerals are made more bioavailable, thereby increasing the nutritional value of the food (Ogbonna et al., 2012; Oghbaei & Prakash, 2016).

## 6 CONCLUSION

Evidence is overwhelming indicating that fermented or germinated foods are superior in nutrients compared to their unfermented or ungerminated counterparts due to activation of endogenous enzymes that degrade antinutritional factors. Antioxidant properties of fermented foods are also elevated compared to their unfermented counterparts due to increased vitamin C and ease of release of different health‐promoting bioactive compounds resulting from weakening of grain matrix. Disruption of food matrices embedding various minerals helps to increase bioavailability of minerals making fermentation and germination key in improving weaning and complementary foods for children especially in regions where diet is predominantly plant based. Postprandial glucose released could be enhanced if fiber is completely disrupted resulting into fast digestive breakdown of starch, while on the other hand when organic acids dominate, fermented food could be recommended for people with diabetes as organic acids lower GI of food. Therefore, optimum fermentation and germination conditions should be determined for each cereal and legume in order to optimize the health and nutrition benefit of these processes.

## CONFLICT OF INTEREST

The authors declare that there is no conflict of interest.

## ETHICAL REVIEW

This study does not involve any human or animal testing.
